# Supplementary material for: ICI-induced Granulomatous Sialadenitis is Responsive to Prednisone
Source: medRxiv. 2026 Jan 26:2026.01.21.26344113. Preprint. [Version 1] doi: 10.64898/2026.01.21.26344113 (PMC12870630; doi:10.64898/2026.01.21.26344113)
Supplement: Supplement 1 [file media-1.pdf]

Supplemental Table and Figure.

**Supplemental Table 1. Clinical testing results at initial and follow-up evaluations**			
Test name	Initial visit	Follow-up visit, 36 days after Initial visit.	Normal Range
ANA Hep-2 substrate S IgG[1]	***Positive, 1:160***	***Positive, 1:160***	<1:80
ENA[2]	***SSA 6.9 units***<br>SSB <0.2 units<br>Anti-RNP <0.2 units<br>Anti-Sm <0.2 units<br>Scl 70 <0.2 units<br>Anti-Jo-1 <0.2 units	***SSA 5.9 units***<br>SSB <0.2 units<br>Anti-RNP <0.2 units<br>Anti-Sm <0.2 units<br>Scl 70 <0.2 units<br>Anti-Jo-1 <0.2 units	<1 units
Ro52-LIPS	***2.6*10^6 RLU[3]***	***1.6*10^6 RLU***	<10^4 RLU
Ro60-LIPS	<10^4 RLU	<10^4 RLU	<10^4 RLU
Quantitative immunoglobulins	IgG 1668 mg/dL<br>IgA 410 mg/dL<br>IgM 89 mg/dL	IgG 1441 mg/dL<br>IgA 380 mg/dL<br>IgM 80 mg/dL	IgG 540-1822 mg/dL<br>IgA 63-484 mg/dL<br>IgM 22-240 mg/dL
C3 complement	135 mg/dL	126 mg/dL	82-185 mg/dL
C4 complement	35 mg/dL	31 mg/dL	15-53 mg/dL
RF[4]	<10 units	<10 units	<10 units
ACE[5]	50.6 units	Not performed	3-52 units/L
Schirmer test without anesthesia	***Right eye 4mm/5 min***<br>***Left eye 3mm/5 min***	***Right eye 5mm/5 min***<br>Left eye 7mm/5min	Right eye 6 mm/5min<br>Left eye 6 mm/5min
Van Bijsterveld[6]	Right eye 1<br>Left eye 0	Not performed	Right eye <4<br>Left eye <4
Unstimulated whole saliva flow rate	***0 mL/15 min***	3.60 mL/15 min	>3.0 mL/min
Minor salivary gland Focus score	7	3	0

Bolded and italicized values indicate they fall outside the normal range. *Abbreviations.* Anti-nuclear antibody Hep-2 substrate S immunoglobulin G; 2. Extractable Nuclear Antigens; 3. Relative Light Units; 4. Rheumatoid factor; 5. Angiotensin converting enzyme; 6. Corneal staining.
