# Supplementary figures and images for: ICI-induced Granulomatous Sialadenitis is Responsive to Prednisone
Source: medRxiv. 2026 Jan 26:2026.01.21.26344113. Preprint. [Version 1] doi: 10.64898/2026.01.21.26344113 (PMC12870630; doi:10.64898/2026.01.21.26344113)

(A)

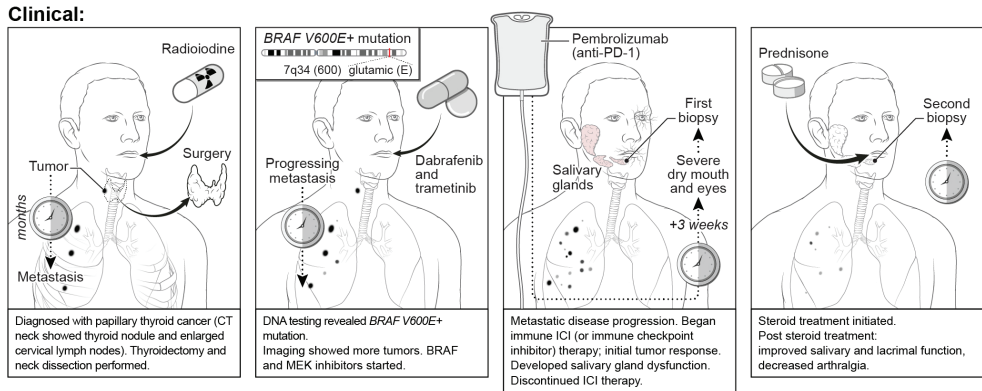

(B)

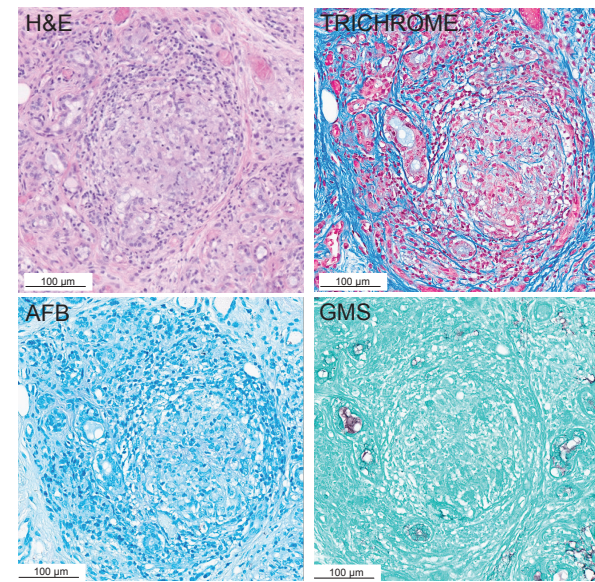

(C)

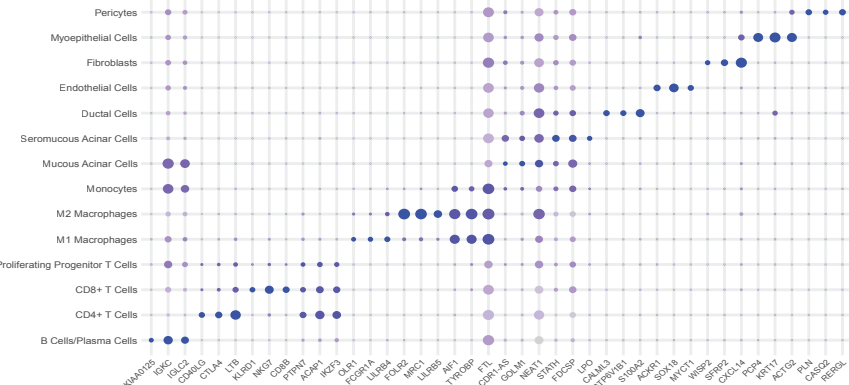

(D)

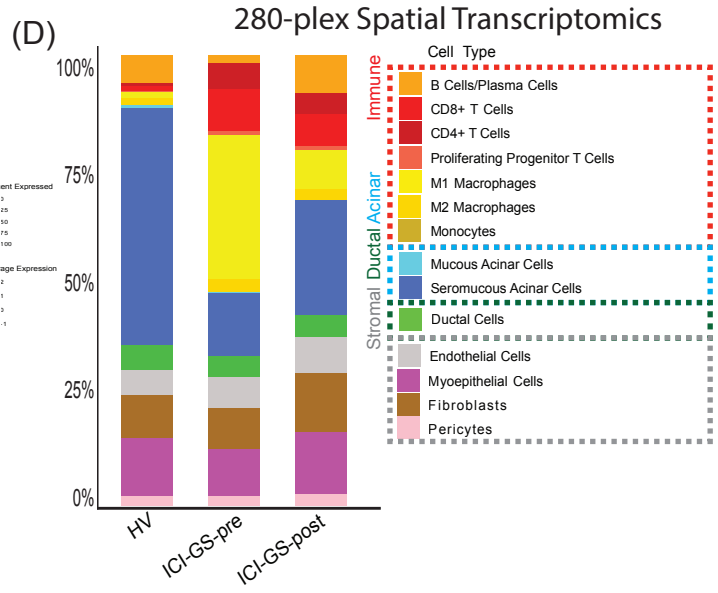

(E)

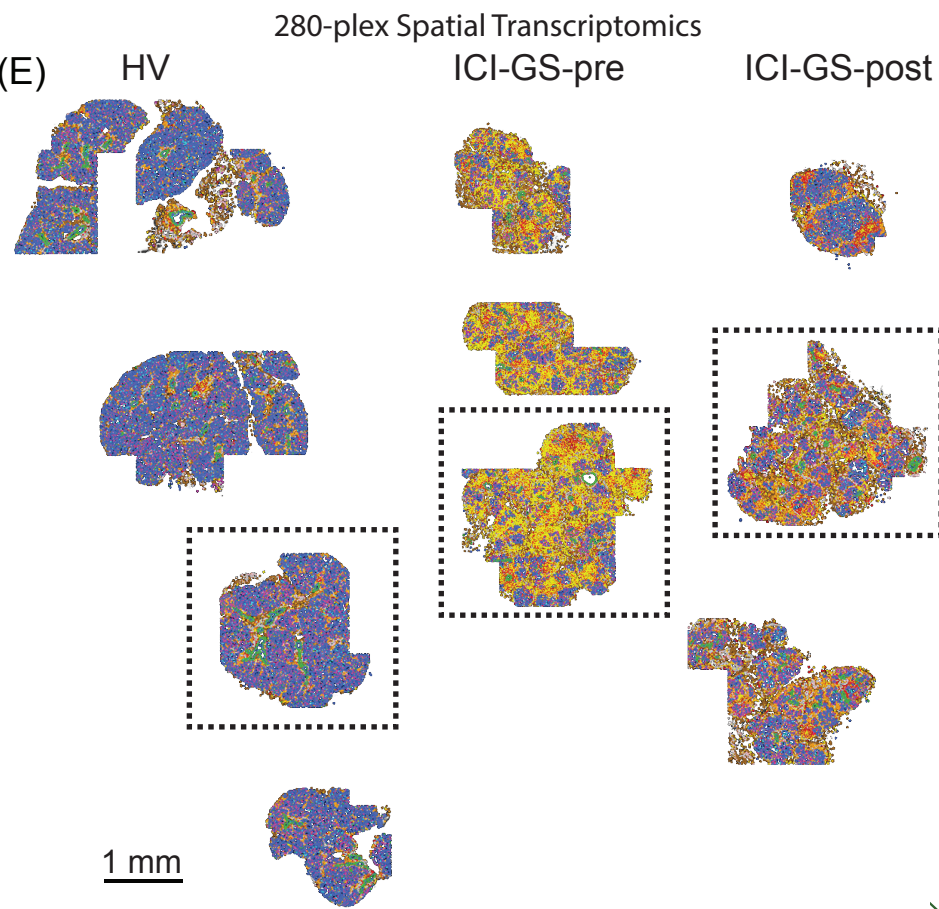

(F)

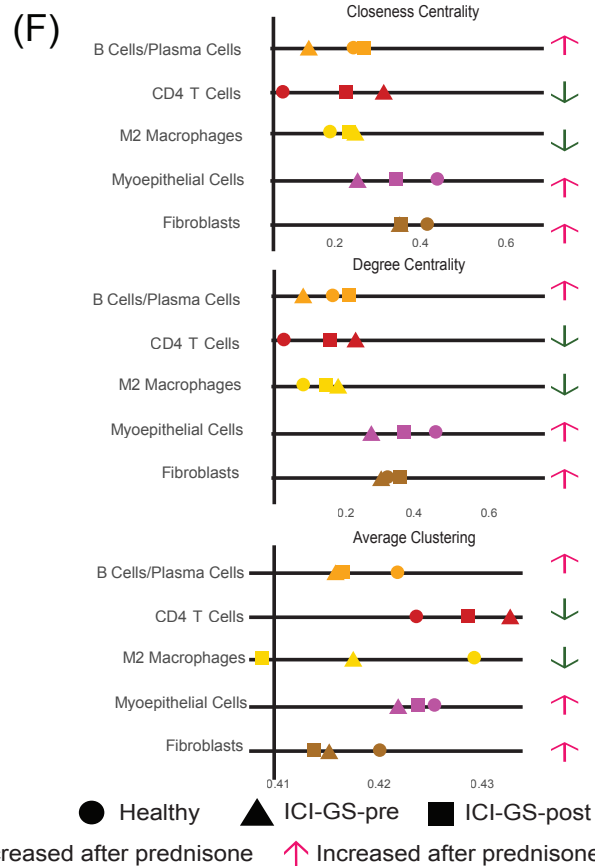

Supplement: Supplement 2 [file media-2.pdf]
