# Supplementary material for: ICI-induced Granulomatous Sialadenitis is Responsive to Prednisone
Source: medRxiv. 2026 Jan 26:2026.01.21.26344113. Preprint. [Version 1] doi: 10.64898/2026.01.21.26344113 (PMC12870630; doi:10.64898/2026.01.21.26344113)
Supplement: Supplement 4 [file media-4.pdf]

Supplemental Table 2. Clinical characteristics of the single cell and spatial transcriptomic data						
**Spit Number***	**Sex**	**Race**	**Ethnicity**	**Histopathological Interpretation**	**Focus Score**	**Case**
3717	Female	White	Not Latino or Hispanic	Mild Chronic Sialadenitis	0	0
3735	Female	White	Latino or Hispanic	Normal	0	0
3743	Female	White	Not Latino or Hispanic	Mild Chronic Sialadenitis	0	0
3632	Female	White	Not Latino or Hispanic	Mild Chronic Sialadenitis	0	0
3633	Female	White	Not Latino or Hispanic	Mild Chronic Sialadenitis	0	0
3437	Female	Black or African American	Not Latino or Hispanic	Mild Chronic Sialadenitis	0	0
3741_pre**	Male	White	Not Latino or Hispanic	Severe Chronic Sialadenitis	7	1
3741_post**	Male	White	Not Latino or Hispanic	Mild Chronic Sialadenitis	3	1

***** - Spit Numbers are unknown to anyone outside the research group.

**** -** Case had biopsies taken for clinical purposes before (“pre”) and after (“post”) effective prednisone taper.
